# Plasma microRNA biomarkers for detection of mild cognitive impairment

**DOI:** 10.18632/aging.100486

**Published:** 2012-09-19

**Authors:** Kira S. Sheinerman, Vladimir G. Tsivinsky, Fiona Crawford, Michael J. Mullan, Laila Abdullah, Samuil R. Umansky

**Affiliations:** ^1^ DiamiR, LLC, Princeton, NJ 08540, USA; ^2^ Roskamp Institute, Sarasota, FL 34243, USA

**Keywords:** microRNA, Mild Cognitive Impairment (MCI), Alzheimer's disease, neurodegeneration, aging, brain, synapse, neurite

## Abstract

Early stages of many neurodegenerative diseases, such as Alzheimer's disease, vascular and frontotemporal dementia, and Parkinson's disease, are frequently associated with Mild Cognitive Impairment (MCI). A minimally invasive screening test for early detection of MCI may be used to select optimal patient groups in clinical trials, to monitor disease progression and response to treatment, and to better plan patient clinical care. Here, we examined the feasibility of using pairs of brain-enriched plasma microRNA (miRNA), at least one of which is enriched in synapses and neurites, as biomarkers that could differentiate patients with MCI from age-matched controls. The identified biomarker pairs fall into two sets: the “miR-132 family” (miR-128/miR-491-5p, miR-132/miR-491-5p and mir-874/miR-491-5p) and the “miR-134 family” (miR-134/miR-370, miR-323-3p/miR-370 and miR-382/miR-370). The area under the Receiver-Operating Characteristic curve for the differentiation of MCI from controls using these biomarker pairs is 0.91-0.95, with sensitivity and specificity at 79%-100% (miR-132 family) and 79%-95% (miR-134 family), and p < 0.001. In a separate longitudinal study, the identified miRNA biomarker pairs successfully detected MCI in majority of patients at asymptomatic stage 1-5 years prior to clinical diagnosis. The reported biomarker pairs also appear useful for detecting age-related brain changes. Further testing in a larger study is necessary for validation of these results.

## INTRODUCTION

Neurodegenerative diseases comprise a large group of pathologies caused by metabolic changes in brain cells, loss of synapses and other compartments of neurons, and, ultimately, neuronal death [1]. Due to increased lifespan, neurodegenerative diseases, such as Alzheimer's Disease (AD), Parkinson's Disease (PD), Huntington Disease, vascular dementia and others, have become very common in developed countries. 13.9% of people age 71 and older in the United States have dementia [2]. Currently, an estimated 5.4 million people have AD in the US alone [2]. A brain's ability to compensate for the dysfunction and loss of neurons, occurring over a long period of time, results in late clinical manifestation of symptoms of AD and other dementias. At late stages of neurodegeneration, serious morphologic changes in the brain, including a massive loss of neurons, have already occurred, and, as a consequence, successful pharmacological intervention is not feasible. Thus, diagnostic methods based on detection of early events in the development of AD and of other dementias are highly desirable.

Mild cognitive impairment (MCI) is usually defined as an intermediate state between normal aging, and AD and other dementias, representing the first stage when clinical symptoms become evident [3-5]. On average, MCI patients convert to dementia at a rate of 10-15% annually [5,6]. Currently, the disease progression of MCI patients cannot be reliably predicted. First, up to 40% of MCI patients revert to normal status [7,8], and autopsy studies demonstrate that a substantial percentage of MCI patients do not develop AD pathology [9,10]. Second, approximately 20% of MCI patients, who progress to dementia, are diagnosed with neurodegenerative diseases other than AD, such as vascular, Lewy body, Huntington, Parkinson, and other dementias [9,11]. Third, disease progression varies from slow to intermediate to rapid [12]. Moreover, MCI is not a homogeneous pathology and is currently described as two clinical conditions - with amnestic symptoms (aMCI) and without amnestic symptoms [8,13]. Some publications have reported that aMCI converts to dementia more frequently [14,15]. However, other authors have not found significant difference in the conversion rate for the two MCI forms [16,17].

Currently, diagnosis of AD and other forms of dementia is based on analysis of the patient's cognitive function. Amyloid plaques between neurons, neurofibrillary tau-tangles, and an overall shrinkage of the brain tissue are the hallmarks of AD, and there have been many attempts to develop diagnostic tests based on these phenomena. Recently published data have demonstrated high sensitivity of AD detection by measuring concentrations of the three protein biomarkers in the cerebrospinal fluid (CSF): beta-amyloid protein 1-42, total tau protein, and phosphorylated tau181P protein [18,19]. However, the invasiveness of the CSF collection procedure makes such assays challenging for everyday clinical use. New imaging techniques, including PET scan for *in vivo* detection of beta-amyloid deposition, are becoming more sensitive and specific but are not suitable for first line screening [20-22]. Several groups have reported encouraging early data on the development of blood assays for AD diagnosis based on analysis of a large number of proteins or antibodies in human blood [23-25].

Neurodegenerative diseases are characterized by neuronal death in specific areas of the brain, for example, hippocampus and cortex for AD, midbrain for PD, frontal and temporal lobes for frontotemporal dementia. However, loss of neurons is a relatively late event in the progression of neurodegenerative diseases that is typically preceded by metabolic changes, such as formation of beta-amyloid plaques and tau protein tangles in AD [1], followed by synaptic dysfunction, synaptic loss, neurite retraction, and the appearance of other abnormalities, such as axonal transport defects [26-29]. Numerous studies are devoted to description of axon destruction with shedding of membrane-enclosed “axosomes”, axon, dendrite and spine pruning, and disassembly of synapses [30-33]. Thus, different processes are characteristic of early and late stages of neurodegeneration and different molecular tests may be needed for early detection of the pathology and monitoring of the pathology progression versus diagnosis and monitoring of a late stage disease.

The present study evaluates the hypothesis that neurite and synapse destruction, which are pathologic processes characteristic of early stages of AD, other neurodegenerative diseases, and MCI syndrome in general, can be detected *in vitro* by quantitative analysis of brain-enriched cell-free miRNA in the blood. MicroRNA (miRNA) is a class of non-coding RNA, whose final product is an approximately 22 nt functional RNA molecule. They play important roles in the regulation of target genes by binding to complementary regions of messenger transcripts and repressing their translation or regulating degradation [34,35]. Thus, miRNA are important epigenetic regulators of numerous cellular processes [35-37]. Many of miRNA are specific to or are over-expressed in certain organs/tissues/cells [38-41]. Some miRNA, including those that are cell-specific, are also enriched in certain cellular compartments, particularly in axons, dendrites and synapses [42-46]. Changes in expression of some miRNA were found in neurons of patients with AD and other neurodegenerative diseases [47-49], as well as in animal models of AD [50,51]. Importantly, cell-free miRNA have been shown to be stable in blood samples [52].

Our approach for developing a non-invasive assay for detection of MCI is based on analysis of levels of brain-enriched miRNA, including neurite- and synapse-enriched miRNA, in plasma and identification of miRNA biomarker pairs capable of successfully differentiating MCI patients from aged-matched controls.

## RESULTS

### Selection of miRNA for pilot study

Two approaches are frequently used for the selection of promising miRNA biomarkers for detection of various cancers and other diseases. The first approach is based on analysis of hundreds of miRNA using miRNA arrays with subsequent validation of potential biomarkers by RT-PCR. In spite of an obvious advantage of this approach (i.e., the analysis of huge miRNA numbers), its disadvantages, namely lower sensitivity and higher variability, make it less suitable for the analysis of cell-free circulating miRNA in plasma or serum: (i) concentrations of many miRNA in plasma are low, and (ii) dramatic changes in miRNA levels should not be expected for a chronic pathology. The second approach is based on analysis of miRNA, whose expression level changes due to a pathology development. This approach also has certain limitations due to potential involvement of the same miRNA in diseases of various organs and because higher expression of miRNA in an affected organ is not necessarily accompanied by an increase in its plasma level [53,54]. In this study we selected the initial pool of miRNA among brain- and neuron-enriched miRNA, suggesting that variations of their concentrations in plasma, if any, are most likely caused by changes in neurons and not in other cell types or organs. Since MCI and early stages of AD are associated with neurite and synapse destruction, we included in the study miRNA, which are not only enriched in neurons but are also known to be present in neurite and synapses [38-46] and involved in neurite- and synapse-associated processes (The miR-Ontology Data Base: http://ferrolab.dmi.unict.it/miro/), suggesting that axon, dendrite and spine pruning and synaptic loss can lead to appearance of these miRNA in the extracellular space and ultimately in the bloodstream. 32 miRNA (Table 1) were selected for the pilot study based on the criteria described above and analyzed by individual RT-PCR, currently the most sensitive and the least variable technique.

**Table 1:** List of miRNA tested in the pilot study (Highlighted are miRNA selected as potential biomarkers for further analysis)

Number	MicroRNA
1	has-miR-7
2	has-miR-9
3	has-miR-9*
4	has-miR-98
5	has-miR-124
6	has-miR-125b
7	has-miR-127-3p
8	has-miR-128
9	has-miR-132
10	has-miR-134
11	has-miR-137
12	has-miR-138
13	has-miR-149
14	has-miR-181a
15	has-miR-181b
16	has-miR-181a*
17	has-miR-218
18	has-miR-323-3p
19	has-miR-330-3p
20	has-miR-370
21	has-miR-382
22	has-miR-383
23	has-miR-409-3p
24	has-miR-433
25	has-miR-485-3p
26	has-miR-487b
27	has-miR-491-5p
28	has-miR-539
29	has-miR-770-5p
30	has-miR-874
31	has-miR-935
32	has-miR-939

Pilot study for selecting promising miRNA biomarkers. The concentrations of miRNA were measured in plasma samples of MCI and age-matched donors with normal cognitive function (Table 2), 10 samples in each group, by RT-PCR. miRNA with low (mean Ct>36) or undetectable plasma concentrations were excluded from the analysis. The ratios of levels of all possible miRNA pairs (2^−ΔCt^) were calculated using a software algorithm developed at DiamiR (see Supplemental Materials). Thirteen miRNA, miR-7, miR-125b, mir-128, miR-132, miR-134, miR-323-3p, miR-382, miR-874, miR-9, miR-127-3p, miR-181a, miR-370, and miR-491-5p, formed pairs differentiating MCI from age-matched controls with p<0.05; these miRNA were selected for further analysis.

**Table 2:** Demographics of plasma donors

Clinical Diagnosis	Number	Age		Sex	MMSE
		Mean	Range	Male/Female	(mean ± SD)
**Pilot Study**					
***Age matched controls (AMC)***	10	77.4	71-85	5/5	28.9 ± 1.1
***Mild cognitive impairment (MCI)***	10	81.7	75-87	5/5	28.1 ± 1.4
**Main Study**					
***Young control (CY)***	20	36.5	21-50	11/9	29.7 ± 2.6
***AMC***	20	80.2	76-86	12/8	29.2 ± 1.3
***MCI***	20	79.9	72-89	15/5	25.8 ± 3.5
***AD***	20	76.9	63-89	13/7	20.8 ± 8.7
**Longitudinal study**	19	77.0	73-84	10/9	28.8 ± 1.3

### Feasibility study for differentiation of MCI and AD from Age-Matched Controls

**Figure 1:**
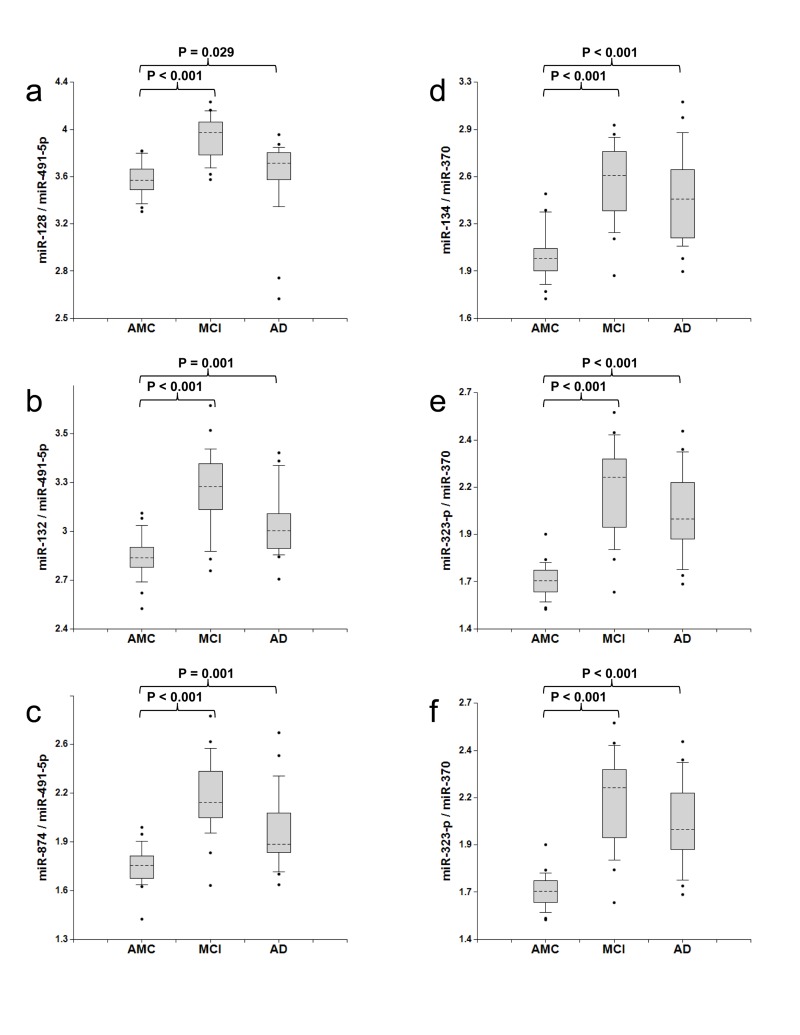
Ratios of miRNA levels (biomarker pairs) in plasma of age-matched controls, MCI, and AD patients The concentrations of miRNA in plasma samples of MCI and AD patients, and age-matched donors with normal cognitive function, 20 samples in each group, were measured by RT-PCR and the ratios of various miRNA were calculated as 2^−ΔCt^ × 100. Here and in other figures with box and whisker plots the results are presented in the Log10 scale. The upper and lower limits of the boxes and the lines inside the boxes indicate the 75th and 25th percentiles and the median, respectively. The upper and lower horizontal bars denote the 90th and 10th percentiles, respectively. The points indicate assay values located outside of 80% data. AMC: age-matches controls; MCI: MCI patients; AD: AD patients.

**Figure 2:**
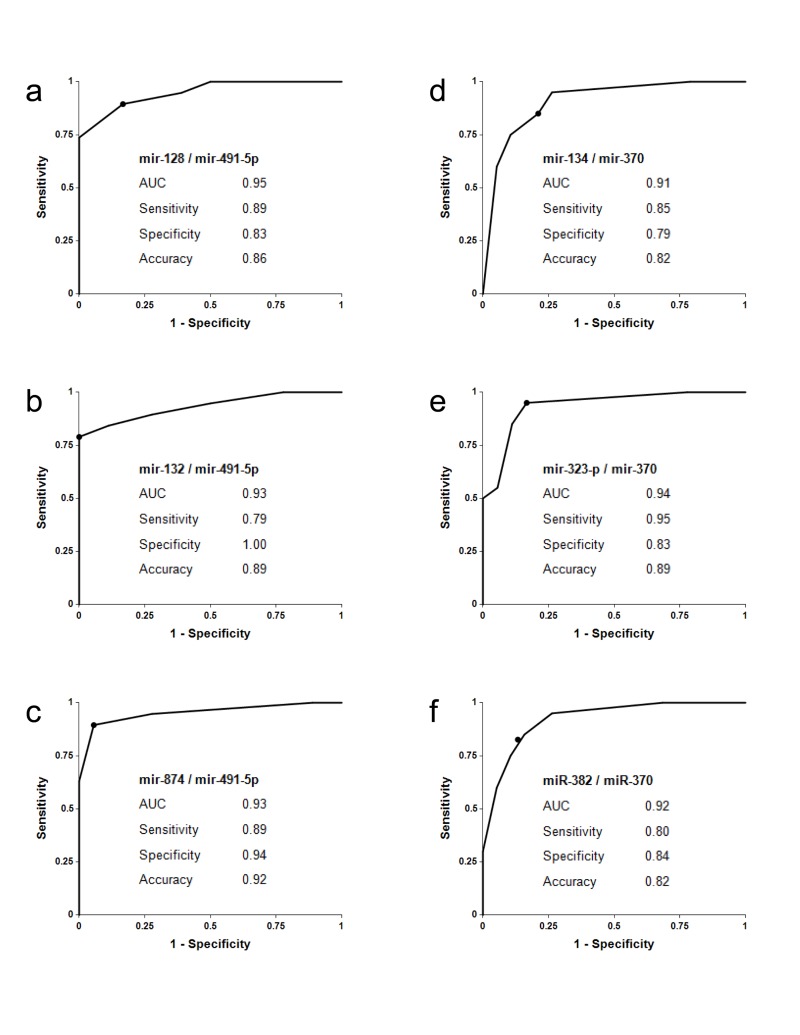
Receiver-Operating Characteristic (ROC) curve analysis of differentiation between MCI patients and age-matched controls obtained with different biomarker pairs The areas under the ROC curve (AUC) are reported. Sensitivity, specificity and accuracy for each biomarker/normalizer pair are calculated for the “cutoff” point (indicated as a dot on each plot); the cutoff point is the ratio of paired miRNA, at which a sample is equally likely to belong to the AMC and the MCI groups (see Supplementary materials for more details).

The concentrations of 13 miRNA selected in the pilot study were determined by the single target TaqMan® miRNA qRT-PCR assay (Applied Biosystems) in the plasma samples of amnestic MCI patients, AD patients and age-matched donors, 20 samples in each group (Table 2). The ratios of levels of all possible miRNA pairs were calculated. The data obtained in this set of experiments are reported in Fig. 1 and S1. Receiver-Operating Characteristic (ROC) curves for miRNA pairs with the highest sensitivity and specificity are presented in Fig. 2.

Biomarker pairs miR-128/miR-491-5p, miR-132/miR-491-5p and mir-874/miR-491-5p (Set 1) differentiated MCI from age-matched control with 79%-89% sensitivity and 83%-100% specificity (Fig. 1a-c and 2a-c). The area under the ROC curve (AUC) for miR-128/miR-491-5p, miR-132/miR-491-5p and miR-874/miR-491-5p is 0.95, 0.93 and 0.95, respectively. In addition, biomarker pairs miR-134/miR-370, miR-323-3p/miR-370 and miR-382/miR-370 (Set 2) demonstrated 80%-95% sensitivity and 79-84% specificity (Fig. 1d-f and 2d-f). AUC for miR-134/miR-370, miR-323-3p/miR-370 and miR-382/miR-370 are 0.91, 0.94 and 0.92, respectively.

**Figure 3:**
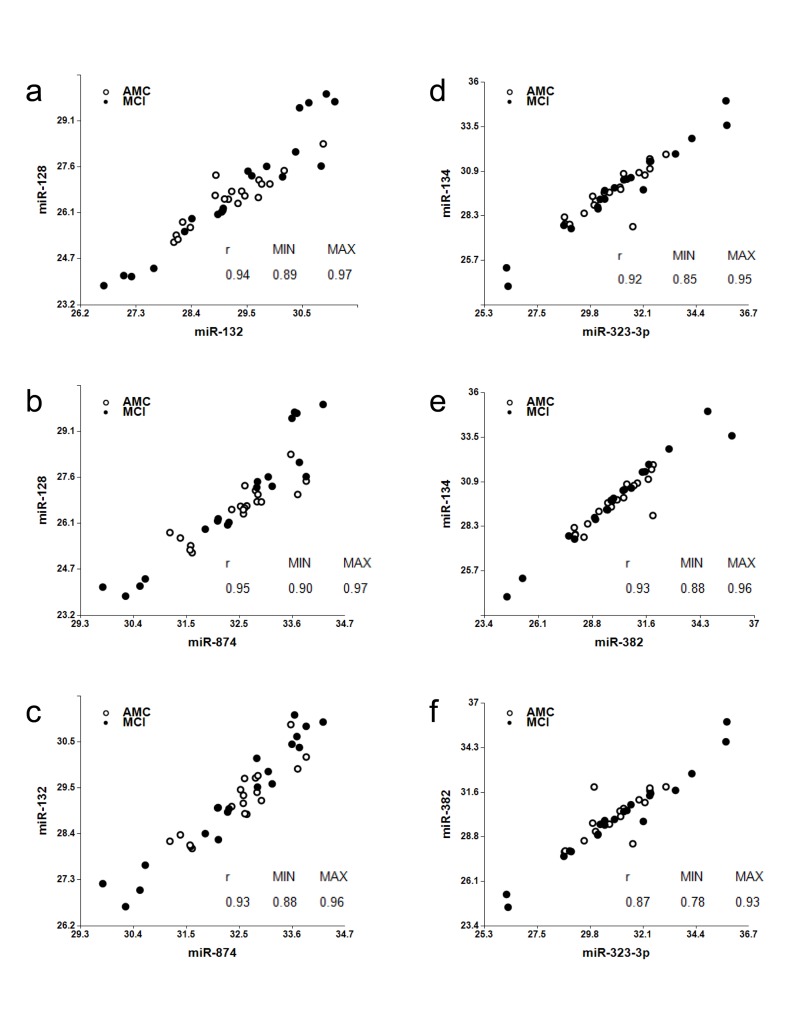
Analysis of associations among miR-128, miR-132, and miR-874 (“miR-132 family”); and miR-134, miR-323-3p and miR-382 (“miR-134 family”) Spearman's rank correlation coefficients r along with 95% confidence intervals (MIN & MAX) are shown.

Each biomarker Set 1 and 2 includes three different miRNA (numerators) paired with the same miRNA (denominator): miR-128, miR-132 and miR-874 are paired with miR-491-5p and miR-134, miR-323-3p and miR-382 are paired with miR-370. miR-128, miR-132 and miR-134 are located in neurites and synapses [42-46]. miR-323-3p and miR382 are enriched in synaptoneurosomes of rat cortex and hippocampus [55]. The predicted targets of miR-874 indicate its involvement in axonogenesis, neurotransmitter secretion, dendrite morphogenesis, synaptogenesis, synaptic transmission and synaptic vesicle exocytosis [The miR-Ontology Data Base: http://ferrolab.dmi. unict.it/miro/]. Thus, each biomarker pair includes a neurite/synapse-enriched miRNA. A correlation analysis shown in Fig. 3 demonstrates that miR-128, miR-132 and miR-874 form one family of biomarkers (miR-132 family) (Spearman test r values in the pair comparison are in the 0.93-0.95 range) and miR-134, miR-323-3p and miR-382 form another family of biomarkers (miR-134 family) (Spearman test r values in the pair comparison are in the 0.87-0.93 range).

Biomarker Set 1 and Set 2 also differentiated AD dementia from the age-matched control with p < 0.05, which is not surprising, since about 50% MCI patients progress to AD; however, these biomarkers did not distinguish AD from MCI, and moreover, the overlap between the distributions of biomarkers for AD and age-matched control was greater than the overlap for MCI and age-matched control (Fig. 1). Two factors may help explain this outcome: (i) as numerous synapses and neuritis are destroyed during earlier stages of the disease, the total amount of excreted synapse/neurite miRNA decreases in later stages of AD, and (ii) in later stages of AD, concentrations of other brain-enriched miRNA (denominator in a biomarker pair) in blood may increase due to their presence in neuronal compartments, glial cells or brain areas, which are involved in the pathology progression.

Supplemental Figure S1 summarizes the results obtained for other miRNA pairs tested for MCI differentiation from age-matched control. These miRNA pairs detect smaller sub-groups of MCI and further studies are necessary to address the question of whether they can be used for detection of particular MCI subsets and for prediction of the disease outcome Retrospective longitudinal study of MCI development in elderly patients with normal cognitive function at enrollment.

The three biomarker pairs of the miR-132 family (Set 1) have shown overall the highest sensitivity and specificity in differentiating MCI from the age-matched control (Fig. 1, 2). These biomarker pairs were, therefore, used to analyze the development of MCI in elderly patients with initially normal cognitive function, recruited in a small longitudinal study at the Roskamp Institute in Florida. Subjects with normal cognitive functions who were at least 70 years old were enrolled and followed for 2-5 years with cognitive assessment and regular collection of plasma. In the course of the study, some subjects remained cognitively normal, while others progressed to MCI. The plasma samples from the 19 subjects, 10 of whom progressed to MCI, were used for miRNA extraction and analysis. In an effort to minimize the effect that a prolonged storage could have had on quality of the samples, patients were classified disease-positive only if in two samples collected at consecutive time points, the concentrations of at least two of the three biomarker pairs, miR-128/miR-491-5p, miR-132/miR-491-5p, and miR-874/miR-491-5p, were higher than the cutoffs determined in the previous experiment (Fig. 2); i.e. if the positive diagnosis made based on the first sample was confirmed using the blood sample collected from the same patient during the next visit. The data, reported in Table 3, demonstrate that in 7 of the 10 subjects who progressed to MCI (patients 10, 12-16, and 19) the increase in plasma levels of miRNA biomarkers is detectable at asymptomatic disease stage, preceding MCI diagnosis by 6 to 61 months. Among the nine patients who remained MCI free, none were classified disease-positive by our assay according to criteria described above.

**Table 3:** Clinical and miRNA-based diagnosis of MCI in elderly subjects with normal cognitive function at the time of enrollment over the course of 2-5 years

Patient	Clinical diagnosis	Time of clinical diagnosis (number of months past enrollment)	miRNA-based diagnosis	Time of miRNA-based diagnosis (number of months past enrollment)	Number of months the miRNA-based diagnosis preceded the clinical diagnosis
C-1	Normal	NA	Normal	NA	NA
C-2	Normal	NA	Normal	NA	NA
C-3	Normal	NA	Normal	NA	NA
C-4	Normal	NA	Normal	NA	NA
C-5	Normal	NA	Normal	NA	NA
C-6	Normal	NA	Normal	NA	NA
C-7	Normal	NA	Normal	NA	NA
C-8	Normal	NA	Normal	NA	NA
C-9	Normal	NA	Normal	NA	NA
MCI-1	MCI	18	MCI	0	18
MCI-2	MCI	33	Normal	NA	NA
MCI-3	MCI	12	MCI	0	12
MCI-4	MCI	23	MCI	0	23
MCI-5	MCI	6	MCI	0	6
MCI-6	MCI	19	MCI	0	19
MCI-7	MCI	61	MCI	0	61
MCI-8	MCI	19	Normal	NA	NA
MCI-9	MCI	16	Normal	NA	NA
MCI-10	MCI	28	MCI	0	28

NA- Not Applicable

### Analysis of normal brain aging with selected miRNA biomarker pairs

**Figure 4:**
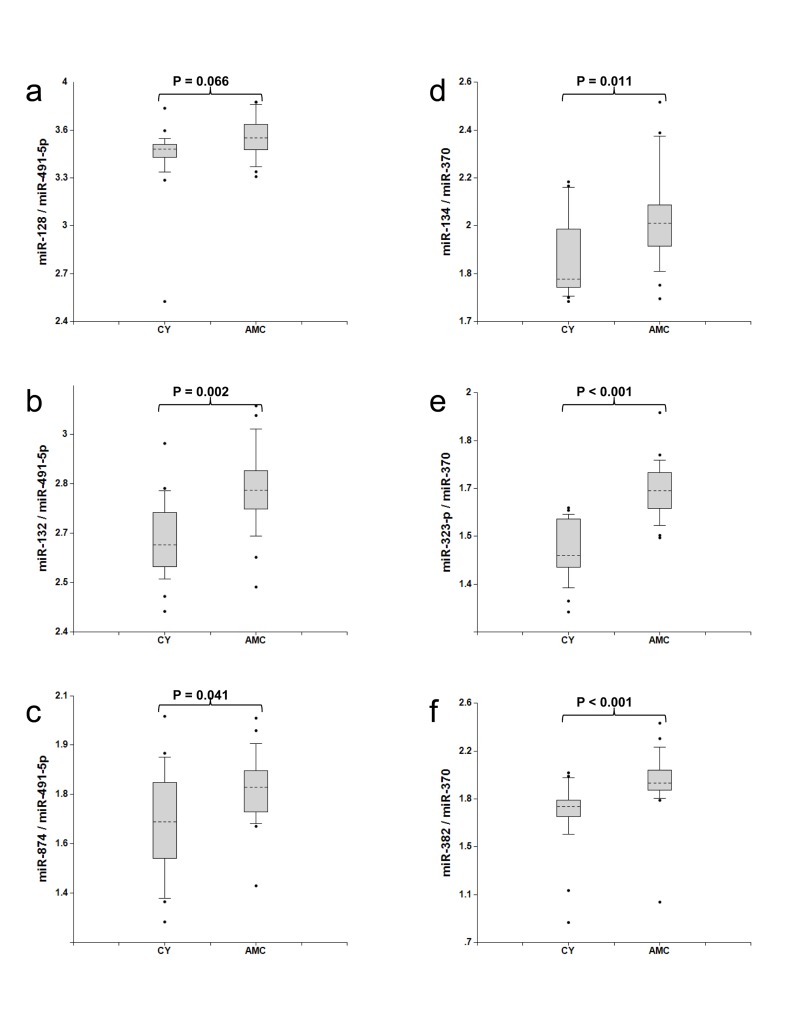
Comparison of miRNA biomarker pairs in plasma of Group 1 (30-50 years old, “CY”) and Group 2 (70-80 years old, “AMC”) individuals with normal cognitive functions The concentrations of miRNA in plasma samples of Group1 (30-50 years old, CY) and Group2 (70-80 years old, AMC) donors with normal cognitive function, 20 samples in each group, were measured by RT-PCR and the ratio of various miRNA was calculated as 2^−ΔCt^ × 100. See the legend to Fig. 1 for the description of the statistical analysis.

The development of MCI, AD and other neurodegenerative diseases on one hand, and normal aging on the other hand share certain common processes, e.g. neurite and synapse destruction and ultimately neuronal death. In this experiment we analyzed whether normal aging could be detected by the same miRNA biomarker pairs. miRNA concentrations in plasma samples from two groups, each comprised of 20 cognitively normal subjects, Group 1 (21-50 years old, “CY”) and Group 2 (76-86 years old, “AMC”), were measured and compared as described above. The data presented in Fig. 4 (Sets 1 and 2) and Fig. S2 (other miRNA pairs) demonstrate that biomarker levels are higher in the plasma of Group 2, “AMC” subjects compared to Group 1, “CY” subjects (*p*<0.05 to*p*<0.001). Thus, a larger prospective longitudinal analysis of these biomarkers in plasma could potentially provide important information on brain processes associated with normal aging.

## DISCUSSION

The objective of the present study was to search for plasma miRNA biomarkers that can be used to detect MCI. The results obtained in our experiments have demonstrated for the first time that a minimally invasive test based on analysis of cell-free miRNA circulating in plasma could be feasible for detection of MCI, AD and even asymptomatic stages of neurodegeneration. The use of brain-enriched neurites/synapses miRNA enables detection of early pathologic events occurring in neurons. Further, combination of neurite/synapse miRNA with other experimentally selected brain-enriched miRNA significantly increases assay sensitivity and specificity at early stages of the pathology, most likely due to compensation for a number of variables, such as blood supply, changes in blood-brain barrier permeability and others.

Two sets of biomarkers have demonstrated high sensitivity and specificity in differentiating MCI from age-matched controls - the miR-132 and miR-134 families paired with miR-491-5p and miR-370, respectively. Although a relatively small number of patients was used in the feasibility study to identify the efficient miRNA biomarker pairs, the data obtained in the longitudinal study (Table 3) and the study of normal brain aging (Fig. 4 and S2) support the findings. Total of 171 plasma samples were analyzed in the experiments reported here. High correlation among members of miR-134 set, namely miR-134, miR-323-3p and miR-382, can be explained by the fact that these miRNA belong to the same cluster (http://www.diana.pcbi.upenn.edu/cgi-bin/miRGen/v3/Cluster.cgi) and are expressed in the same cell types. Close functional relatedness among members of miR-132 set, namely miR-128, miR-132 and miR-874, has not been described before. It is also interesting to note that miR-132 and miR-134 biomarker sets demonstrate higher sensitivity and specificity when paired with different brain-enriched miRNA. The miR-132 set is a strong match with miR-491-5p, miR-181a, and miR-9, while the miR-134 set demonstrates the strongest differentiation between MCI and age-matched controls when paired with miR-370 and miR-127. Correlation between the two miRNA sets (data not shown) is relatively low (*r* values in the pair comparison Spearman test are in the 0.56-0.79 range) indicating that they possibly reflect distinct pathological processes, or are enriched in different brain areas. A mechanistic explanation for this observation is currently missing, and could be provided by a detailed analysis of expression of all these miRNA in various brain areas and cell types.

It is important to mention that most of elderly patients and age-matched controls, as well as some of young controls had various non-neurological conditions unrelated to MCI. However, since this is expected to be the case in a real-life test application, such a test should be capable of detecting MCI in subjects with accompanying diseases. Thus, only patients with a history of a stroke or other neurologic pathologies were excluded from the present study. We believe that the ability of selected miRNA pairs to differentiate MCI (and AD) from age-matched control in spite of the presence of other pathologies supports our approach to biomarker selection from brain-enriched miRNA. The same consideration applies to the comparison between younger and older groups. Additional larger studies are necessary for further data validation, including a prospective longitudinal study. miR-132 and miR-134 families paired with other brain-enriched miRNA effectively distinguish MCI and AD from age-matched control but do not differentiate MCI and AD from each other. Thus, other biomarkers are necessary for prediction of MCI progression to AD and other dementia. The experiments aimed at detection of the MCI sub-types that will progress to AD dementia are currently in progress at DiamiR. The differentiation of AD from other dementias (vascular, frontotemporal, Lewy bodies, etc.) is another important goal and we hope that analysis of miRNA enriched in different brain areas could be useful for differential diagnosis. Further, there are other brain-enriched miRNA, which were not included in the present study but could be found useful as potential biomarkers in the future. Additional promising miRNA along with those described in the present study could be used for detecting other neurodegenerative diseases and for differential diagnosis.

Early detection of MCI patients by a minimally invasive, screening test may make more invasive and expensive tests for detection of AD and other neurodegenerative diseases more practical, since the latter can be applied to the pre-selected cohorts of patients.

Numerous data demonstrate changes in miRNA expression associated with cellular senescence and *in vivo* aging [56-59]. Li et al. described increase in levels of miR-34a in the brain, peripheral blood mononuclear cells, and plasma during aging in mice [60]. It is intriguing that in our study the miRNA biomarker pairs found to differentiate MCI from age-matched controls can be used to register changes during normal brain aging, suggesting that the approach reported in the present study enables detection of processes common for normal aging and MCI development, e.g. destruction of synapses, and could be helpful in basic neurophysiology research of aging. A larger study with subjects representing various age groups (20-30, 30-40…80-90 y. o.) is necessary for validation of these initial findings.

Recently, the National Institute of Aging and Alzheimer's Association has developed new diagnostic guidelines for AD [61-63]. The guidelines contain updated classification of the AD phases, namely the dementia phase, the symptomatic pre-dementia phase (MCI), and the asymptomatic, preclinical phase of AD (pre-MCI). The new guidelines also provide recommendations for the diagnosis of pre-MCI, MCI and AD dementia and stress the current lack of and a great need for reliable biomarkers, which can be used for detection of MCI and preclinical phases of AD. We believe the current study makes a significant contribution towards this objective.

## MATERIALS AND METHODS

### Plasma samples

The plasma samples used in the present study were collected at the Roskamp Institute Memory Center between 2005 and 2009 under the protocol approved by the Western Institutional Review Board (WIRB). An IRB approved written consent was obtained from each subject recruited in the study and the informed consent process was conducted in accordance with the International Conference on Harmonization (ICH) guidelines. If a subject was not medically capable or legally competent to provide consent for participation in the study, a written consent was obtained from a family member, a legally authorized representative (LAR) or health care surrogate (under 21 CFR 50: exceptions from general requirements for informed consent). An assent was obtained from the participant. Venous blood was collected in EDTA vacutainers (BD Diagnostics), which were immediately centrifuged at 1380 × g for 5 minutes. Samples were maintained at 4°C during the plasma preparation process and aliquoted immediately in 1.5ml Eppendorf tubes for storage at −80°C until further use. The use of the samples in the present study was additionally approved by the WIRB in 2010. The quantity and the type of the samplesused in the present study are as follows (Table 2): Pilot Study: amnestic MCI and age-matched donors (> 70 years old) with normal cognitive function, 10 samples in each group; MCI and AD detection: amnestic MCI patients, AD patients and age-matched donors (> 70 years old), 20 samples in each group; Retrospective longitudinal study: samples from 19 subjects, each subject at least 70 years old and having normal cognitive function at the time of the first plasma collection, multiple samples collected from each subject over the course of 2-5 years; Detection of normal brain aging: samples from 20 subjects, 30-50 years old and normal cognitive function, as well as the samples from 20 subjects, each at least 70 years old and having normal cognitive function, which were used as control in the MCI and AD detection study.

### MCI and AD diagnosis

The age-matched controls (AMC) were either recruited from the Roskamp Institute Memory Clinic screening programs conducted in Tampa and Sarasota, FL or through the Alzheimer's disease Anti-inflammatory Prevention Trial (ADAPT) Tampa, FL site. The subjects from ADAPT underwent a brief neuropsychological assessment at enrollment as described elsewhere to determine cognitively normal status [64]. For all AMC, mini mental status examination (MMSE) was also administered to determine cognitive status. In addition, AMC subjects maintained independent activities of daily living and were free of any active neurological illness, psychiatric disorders, or other medical conditions that would potentially interfere with their cognitive performance. Individuals suspected of having MCI or AD underwent a comprehensive dementia work-up which included physical and neurological examinations, laboratory studies (i.e., CBC, chemistry count, sedimentation rate, vitamin B12 and folic acid levels, thyroid test and syphilis serological test) and neuroimaging (i.e., MRI or CT), as applicable. A more comprehensive neuropsychological assessment was also administered as part of the dementia work-up and consisted of expanded Consortium to Establish a Registry for Alzheimer's Disease (CERAD) battery [65]. Learning and memory functions were evaluated using the CERAD 10-word, 3-trial list learning task and CERAD delayed recall measure and Logical Memory I and II of the Wechsler Memory Scale - Revised [66]. The CERAD Constructional Praxis test and Judgment of Line Orientation Test measured visuospatial ability [67]. Language and/or executive measures included 15-item Boston Naming Test, Animal Fluency, the Control Oral Word Association Test (COWAT; CFL); and the similarities subtest from the Wechsler Adult Intelligence Test - 3rd Revision (WAIS-III) [68]. The Trails A of the Trail Making Test and Digit Symbol from the WAIS-III were utilized to measure visual scanning and processing speed. Set-shifting (an executive ability) was measured using Trails B and the Letter Number Sequencing subtest from the WAIS-III [69]. Following dementia work-up, a consensus team determined cognitive status using published diagnostic criteria. The diagnosis of AD was made using NINCDS-ADRDA [70] and amnestic MCI according to the Petersen criteria [71]. MCI and AD patients as well as control subjects with a known history of a stroke or other neurologic pathologies were excluded from the study.

Plasma RNA extraction and qRT-PCR miRNA analysis miRNA isolation and qRT-PCR analysis of the initial set of 32 miRNA, performed in the course of the Pilot study, were performed by Gene Logic (an Ocimum Biosolutions Company, Gaithersburg, MD, USA) according to the following protocol. RNA was extracted from 250 μl aliquots using mirVanaTM Paris Extraction Kit and protocol (Ambion). 2.5×107 copies of Arabidopsis thaliana miR-159a (ath-mir-159a) were spiked per 0.25 μl plasma after addition of guanidine-containing denaturing solution for evaluating miRNA yield. Single target qRT-PCR was performed using the TaqMan® Reverse Transcription Kit and miRNA-specific stem-loop primers (Applied Biosystems). Final PCR was performed in triplicate using 3.3 μl plasma equivalents. Based on the quantitative measurement of spiked ath-miR-159a, average yield of miRNA from plasma was about 70%.

miRNA isolation and qRT-PCR analysis in all other experiments were performed by Asuragen Inc. (Austin, TX, USA) according to the following protocol. RNA was extracted from 200 μl aliquots using Asuragen's proprietary protocol, which is based on Trizol treatment and silica (Ambiom Glass Fiber Microcolumn) binding. Single target qRT-PCR was performed using the TaqMan® Reverse Transcription Kit and miRNA-specific stem-loop primers (Applied Biosystems). RT step was performed in triplicate and 2 μl plasma equivalents were present in final PCR. The concentrations of the 13 miRNA (8 neurite/synapse miRNA and 5 other brain-enriched miRNA), were determined in the plasma samples of amnestic MCI patients, AD patients and age-matched donors, 20 samples in each group (Table 2). The sample size for this study was determined by a standard formula for a case-control study [72] using power = 0.8, significance level = 0.05 and the ratio of standard deviation to difference between comparison groups set to 1. The ratios of levels of all possible miRNA pairs were calculated.

### Bioinformatics analysis and statistical methods

In addition to biological factors, such as levels of expression, secretion, blood-brain barrier permeability, etc., miRNA yield from plasma may depend on a purification technique. Further, presence of RT-PCR inhibitors in the blood may vary from subject to subject and distort an experimental outcome. Therefore, data normalization becomes an issue of critical importance. Two normalization approaches that are commonly used in miRNA studies include: (i) normalization per the least variable miRNA, such as spiked non-human miRNA or ubiquitous miRNA, whose concentration is expected to be minimally changed by a pathology being analyzed [73], and (ii) normalization based on an experimental search for miRNA pairs, which most effectively differentiate two populations, e.g. pathology versus control [74,75]; ratios of levels of all possible miRNA pairs from the same sample are calculated and the most promising pairs (self-normalizing biomarkers) are selected for further testing and validation. The advantage of the second approach is that in certain cases miRNA, whose concentrations are changed due to a pathology in opposite directions, can be effective in differentiating investigated populations. We use the latter approach and, in addition to brain-enriched miRNA present in neurites and synapses, measure other brain-enriched miRNA to compensate for variations in blood supply, blood/brain barrier permeability, and other brain-specific factors.

All statistical calculations were performed with the use of custom software developed at DiamiR LLC (Princeton, NJ), as described in the Supporting Information (Software, Calculations, Graphical Interface). Mann-Whitney U-tests were used to evaluate significance of differentiation of any two patient groups by various miRNA pairs. Spearman's rank correlation coefficient was calculated to estimate associations between various biomarkers. P-value < 0.05 was considered significant; actual p-values are reported for each experiment. Receiver-Operating Characteristic (ROC) curves were constructed and the area under ROC curves (AUC) was calculated to evaluate sensitivity and specificity of various biomarker sets. The cutoff points on the ROC curves, at which accuracy of MCI detection is maximal, were selected.

## SUPPLEMENTARY MATERIALS


