# Supplementary material for: Plasma microRNA biomarkers for detection of mild cognitive impairment
Source: Aging (Albany NY). 2012 Sep 19;4(9):590–605. doi: 10.18632/aging.100486 (PMC3492224; doi:10.18632/aging.100486)
Supplement: Supplementary file 1 [file aging-04-590-s001.pdf]

Association workgroups on diagnostic guidelines for Alzheimer's disease. Alzheimers Dement. 2011; 7:263-269.

62. Albert MS, DeKosky ST, Dickson D, Dubois B, Feldman HH, Fox NC, Gamst A, Holtzman DM, Jagust WJ, Petersen RC, Snyder PJ, Carrillo MC, Thies B, Phelps CH. The diagnosis of mild cognitive impairment due to Alzheimer's disease: Recommendations from National Institute on Aging-Alzheimer's Association workgroups on diagnostic guidelines for Alzheimer's disease. Alzheimers Dement. 2011; 7:270-279.

63. Sperling RA, Aisen PS, Beckett LA, Bennett DA, Craft S, Fagan AM, Iwatsubo T, Jack CR Jr, Kaye J, Montine TJ, Park DC, Reiman EM, Rowe CC, Siemers E, Stern Y, Yaffe K, et al. Toward defining the preclinical stages of Alzheimer's disease: Recommendations from National Institute on Aging-Alzheimer's Association workgroups on diagnostic guidelines for Alzheimer's disease. Alzheimers Dement. 2011; 7:280-292.

64. Abdullah L, Luis C, Paris D, Mouzon B, Ait-Ghezala G, Keegan AP, Wang D, Crawford F, Mullan M. Serum Abeta levels as predictors of conversion to mild cognitive impairment/Alzheimer disease in an ADAPT subcohort. Mol Med. 2009; 15:432-437.

65. Morris J, Heyman A, Mohs R Hughes JP, van Belle G, Fillenbaum G, Mellits ED, Clark C. The consortium to establish a registry for Alzheimer's disease (CERAD). Part I. Clinical and neuropsychological assessment of Alzheimer's disease. Neurology. 1989; 39:1159-1165.

66. Wechsler D. (1987) Wechsler Memory Scale – Revised. San Antonio, TX: The Psychological Corporation.

67. Benton A, Sivan A, Hamsher K, Varney N, & Spreen O. (1983) Judgment of Line Orientation. Psychological Assessment Resources, Inc., Lutz, FL.

68. Wechsler D. (1997) Wechsler Adult Intelligence Scale – 3rd Edition. San Antonio, TX: The Psychological Corporation.

69. Luis CA, Abdullah L, Ait-Ghezala G, Mouzon B, Keegan AP, Crawford F, Mullan M. Feasibility of Predicting MCI/AD Using Neuropsychological Tests and Serum β-Amyloid. Int. J. Alzheimers Dis. 2011; 2011 Article ID 786264.

70. McKhann G, Drachman D, Folstein M, Katzman R, Price D, Stadlan EM. Clinical diagnosis of Alzheimer's disease: report of the NINCDS-ADRDA Work Group under the auspices of Department of Health and Human Services Task Force on Alzheimer's Disease. Neurology. 1984; 34:939-944.

71. Petersen RC, Smith GE, Waring SC, Ivnik RJ, Tangalos EG, Kokmen E. Mild cognitive impairment: clinical characterization and outcome. Arch Neurol. 1999; 56:303-308.

72. Eng J. Sample Size Estimation: How Many Individuals Should Be Studied? Radiology. 2003; 227:309-313.

73. Peltier HJ, Latham GJ. Normalization of microRNA expression levels in quantitative RT-PCR assays: identification of suitable reference RNA targets in normal and cancerous human solid tissues. RNA. 2008; 14:844-852.

74. Hennessey PT, Sanford T, Choudhary A, Mydlarz WW, Brown D, Adai AT, Ochs MF, Ahrendt SA, Mambo E, Califano JA. Serum microRNA biomarkers for detection of non-small cell lung cancer. PLoS One. 2012; 7:e32307.

75. Matthaei H, Wylie D, Lloyd MB, Dal Molin M, Kemppainen J, Mayo SC, Wolfgang CL, Schulick RD, Langfield L, Andruss BF, Adai AT, Hruban RH, Szafranska-Schwarzbach AE, Maitra A. miRNA Biomarkers in Cyst Fluid Augment the Diagnosis and Management of Pancreatic Cysts. Clin Cancer Res. 2012 Aug 21. [Epub ahead of print].

SUPPLEMENTARY MATERIALS

Software, Calculations, Graphical Interface

Program overview

• The data is imported from Excel spreadsheet using special wizard; no template for submitted data needed. Data can be raw Ct values, or processed, e.g. normalized data;

• Several groups of patients (Target groups) can be specified. Any group can be designated as "Control"; differentiation of all other groups from the Control group can be evaluated;

• ROC parameters are calculated for any miRNA combination, including complex biomarkers, comprising more than one miRNA as numerator, denominator or both;

• Several kinds of customized graphs are displayed, including Points and Box Plots for a set of data, 2D graphs for two markers, ROC graphs for several markers and their combinations, histograms.

Data Flow and Technology

• The software is created in .Net V4.0 as a WinForm application, using C# and VS 2010 / VSS as Development environment. For Math and Statis-tic calculations, MS Excel COM interface is utilized. For histogram support, MathIridium library is used.

• After importing, data converted to internal format and saved for future use in this format, preventing any inadvertent data altering. Along with source data, normalization settings & results, the last status of data analysis (Control and Target groups), are saved. For Markers and Targets, aliases are allowed.

Normalization

Calculations of normalized values is performed according to the following equation:

C = 2-(Ct [miRNA1]-Ct [miRNA2]) * M, where

Ct [miRNA2] – experimentally determined Ct value for a potential denominator;

Ct [miRNA1] – experimentally determined Ct value for a potential numerator;

M is a constant multiplier, chosen, so that to keep the normalized values C in a computationally convenient range, 10 – 1000.

Statistics

Along with regular data set parameters (average, median, standard deviation, finding outliers), some parametric and not-parametric statistics are used:

• Chi-square test with histograms – to define normality of data set;

• T – test (Student) of significance for normally distributed data;

• Pearson correlation coefficient for normally distributed data;
• Mann-Whitney test of significance for other distributions;
• Spearman's (Rank) association coefficient for other distributions.

For calculation of ROC parameters probabilistic approach is used. The calculated parameters include AUC, Sensitivity, Specificity, Accuracy, Matthews correlation coefficient (MCC), F1 score, and combined value as a weighted sum of all parameters.

Conversions
Linear approximation is used for each data set probability integral function. Every sample gets two probability values: to belong to Control, and to belong to Target group. These probability values hold information about a position of a given data point with respect to other points in the dataset and, rather than experimental raw data, are used for statistical analysis (correlation, significance of separation, combination). The use of these probability values allows one to easily perform operations with values, which are all in the range of 0 to 1.0; to conveniently make "once for all" changes in the decision algorithm, such as shifting cut-off points; and to create a complex function of biomarkers, comprising several miRNA pairs, etc.

Decision
Binary decision is based on comparing an actual value and value of cutoff point. In probabilistic approach, we are using probability of the sample to belong to the Target group with probability assigned to cutoff point. "Equilibrium" point, i.e. point where probabilities to belong to Control and Target groups are equal, is selected as the initial value for the cutoff point on ROC curve. Moving this point right or left means creating preference for Specificity over Sensitivity, or vice versa. An important aspect here is that this preference can be introduced for all biomarkers by including one cutoff value in the decision making.

Graphical Interface: a Screenshot

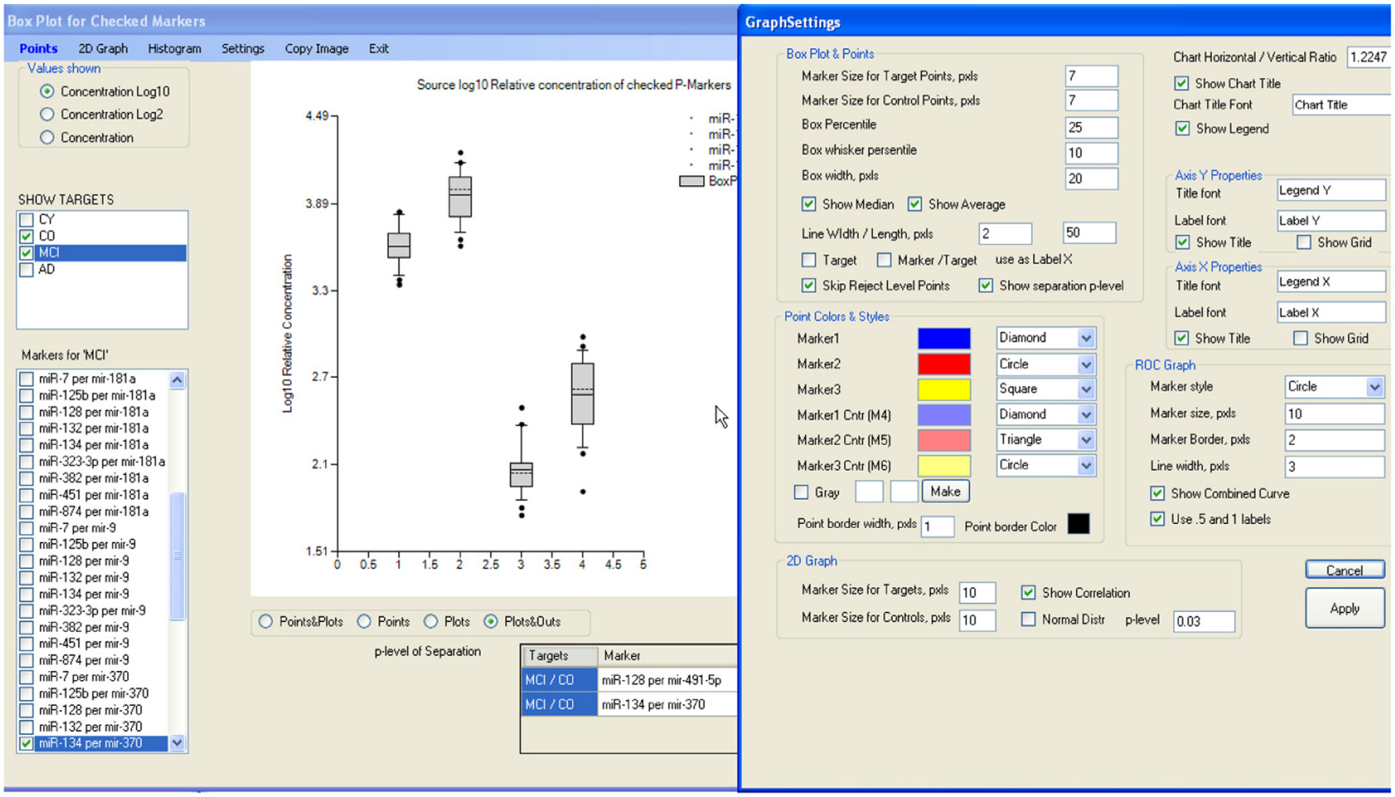

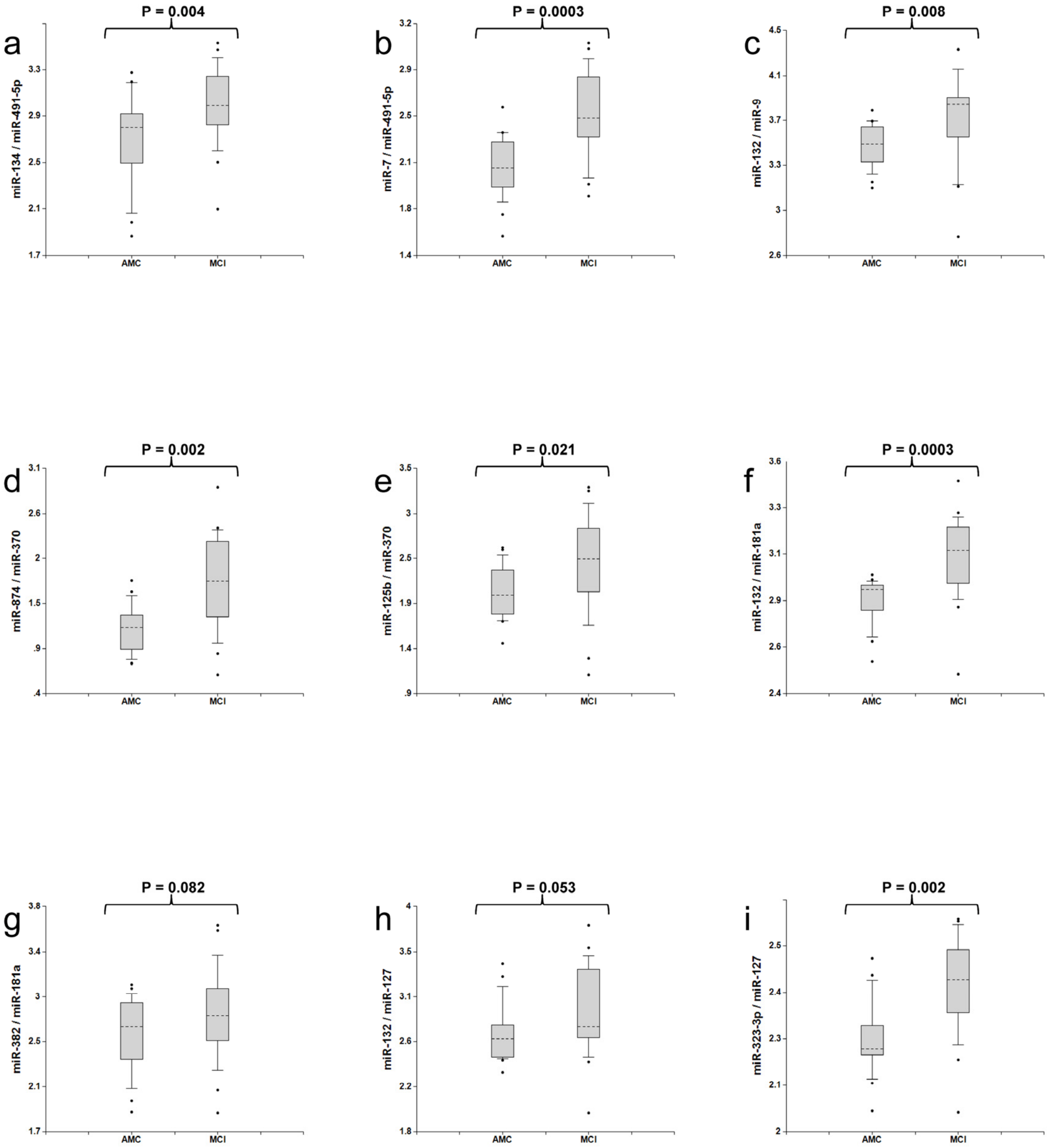

Figure S1: Ratios of miRNA levels (biomarker pairs) in plasma of MCI patients and age-matched controls. The ratios of concentrations of miRNA pairs in plasma samples of MCI and age-matched donors with normal cognitive function, 20 samples in each group, were measured by RT-PCR and the ratios of various miRNA were calculated as $2^{-\Delta Ct}$ x 100. See the Fig. 1 legend for the description of the statistical analysis.

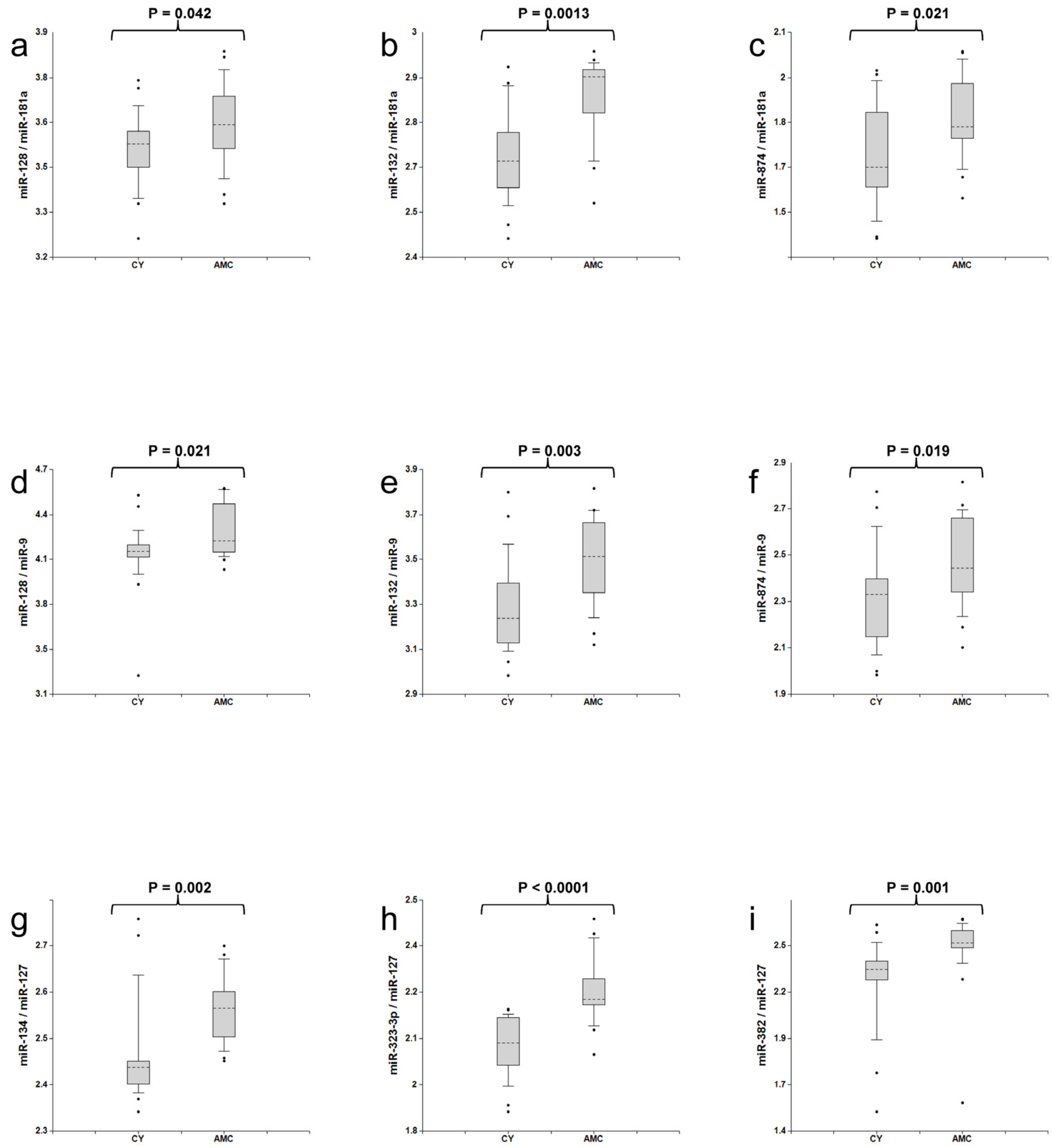

Figure S2. Comparison of miRNA biomarker pairs in plasma of Group 1 (30-50 years old, "CY") and Group 2 (70-80 years old, "AMC") individuals with normal cognitive functions. The ratios of concentrations of miRNA in plasma samples of Group1 (30-50 years old, CY) and Group2 (70-80 years old, AMC) donors with normal cognitive function, 20 samples in each group, were measured by RT-PCR and the ratio of various miRNA was calculated as $2^{-\Delta Ct}$ x 100. See the legend to Fig. 1 for the description of the statistical analysis.
